# Impact of CHIKV Replication on the Global Proteome of *Aedes albopictus* Cells

**DOI:** 10.3390/proteomes10040038

**Published:** 2022-11-10

**Authors:** Ramesh Kumar, Divya Mehta, Sakshi Chaudhary, Debasis Nayak, Sujatha Sunil

**Affiliations:** 1 Vector Borne Diseases Group, International Centre for Genetic Engineering and Biotechnology, New Delhi 110067, India; 2 Department of Biosciences and Biomedical Engineering, Indian Institute of Technology, Indore 453552, India

**Keywords:** chikungunya virus, *Aedes albopictus*, mass spectrometry, pathway analysis, host–pathogen interaction

## Abstract

Arboviruses are some of the important causative agents of mosquito-mediated viral diseases. These viruses are transmitted between vector and host during the blood meal. Upon viral entry, host replication machinery is hijacked, supporting new virus particle production and thereby allowing viral survival in the host. In this process, host proteins interact with viral proteins to either facilitate viral replication, or they may provide antiviral defense mechanisms. In this study, we analyzed the impact of chikungunya virus (CHIKV) infection on the global proteome of Dicer active *Aedes albopictus* cells during the early and late time points of infection. We utilized a bottom-up approach of global proteomics analysis, and we used label-free quantitative mass spectrometry to identify the global protein signatures of *Ae. albopictus* at two different time points upon CHIKV infection. The mass spectrometry data analysis of the early time point revealed that proteins belonging to pathways such as translation, RNA processing, and cellular metabolic processes were less in abundance, whereas those belonging to pathways such as cellular catabolic process and organic substance transport were significantly abundant. At later time points, proteins belonging to pathways such as cellular metabolic processes, primary metabolic process, organonitrogen compound metabolic process, and organic substance metabolic process were found to be decreased in their presence, whereas those belonging to pathways such as RNA processing, gene expression, macromolecule metabolic processing, and nitrogen compound metabolic processing were found to be abundant during CHIKV infection, indicating that modulation in gene expression favoring cell survival occurs at a later time point, suggesting a survival strategy of *Aedes* cells to counter prolonged CHIKV infection.

## 1. Introduction

Amongst the RNA viruses that have garnered public health attention are arboviruses, notorious for their worldwide spread and recurrent outbreaks [1]. Chikungunya virus (CHIKV) is one such arbovirus belonging to the *Togaviridae* family causing chikungunya fever (CHIKF), a major public health concern owing to the prolonged debilitation it leaves in the infected individuals, thereby impacting the nation’s disability-adjusted life years (DALYs) [2]. CHIKF starts with CHIKV infecting the cells such as epithelial, endothelial, fibroblast, and macrophages, and thereafter, the virus spreads to other organs leading to immune response generation mediated by interferons and interleukins [3,4]. The symptoms of fever are generally resolved within a few days; however, in some cases, symptoms of arthralgia were reported even after several months post-acute phase; severe morbidity and mortality have also been reported in some cases [5,6,7].

Historically, CHIKV circulated in sub-Saharan Africa, involving non-human primates as hosts and mosquitos as vectors. Severe deforestation followed by urbanization led to the virus’s emergence, and the first clinical report was reported in Tanzania in 1953 [8]. The infection then spread in and beyond the African region, resulting in the occurrence of three geographically distinct genotypes, namely, West African, Asian, and ECSA with *Ae. aegypti* as the principal vector [1,9,10]. Post-2005, a huge epidemic in the Indian Ocean Islands led to the expansion of the ECSA genotype into sub-lineages such as the Indian Ocean Lineage (IOL). One of the reasons for the occurrence of the epidemic was the successful incorporation of a point mutation in envelope protein 1 (E1) of the virus, which allowed the virus to amplify better in the atypical vector *Aedes albopictus* [11,12,13]. As *Ae. albopictus* was able to better survive in cold temperatures and rural areas, the vector caused the spread of CHIKV to temperate regions from Asia to Europe, America, and Africa [14,15]. The physiological and molecular mechanisms that might be involved in the enhanced vectoral capacity of *Ae. albopictus* has been reported in several studies in the recent past [16,17,18]. These findings have been made possible owing to better physiological and molecular understanding of the vector.

One of the major attributes of the adaptability of *Aedes* geographically, physiologically, as well as in its vectoral capacity is the complexity of its genome. Having one of the largest genome sizes for any mosquito species [19,20], the proteome of this mosquito is expected to be even more complex. Earlier attempts to decipher the proteins of the Dicer-deficient *Ae. albopictus* cell line as well as that of *Ae. aegypti* were focused on canonical proteins [21,22,23]. In recent times, in addition to the traditional top-down approaches to identifying proteins, with the development of sophisticated high throughput technologies such as isotope labeling and label-free mass spectrometry, it is possible to study the global impact of the virus on host proteome and pathways, which play a crucial role in the protection of host from the virus as well as promotion of virus survival [24,25,26,27,28]. Needless to say, both these approaches have provided immense information regarding *Aedes* physiology and insect complexity. The bottom-up approach that involves digesting the proteins/protein/mixture with trypsin and running the peptides in electrophoresis, followed by mass spectrometry analysis, has been widely used in studies related to *Aedes*, such as understanding proteome modulation, identification of anti-viral factors, insecticidal resistance [29], the influence of viral dissemination [30], and the interacting partners of *Aedes* salivary gland proteins in human [31]. The top-down approach that involves ionization of intact protein, which are then analyzed via mass spectrometry and help in identifying proteoforms and post-translation proteolytic processing products commonly called proteoforms, is yet to gain popularity concerning the deciphering of *Aedes* biology.

The present study is an attempt to expand the existing knowledge of the interactions between *Ae. albopictus* and CHIKV during infection by studying global proteome changes in *Ae. albopictus* cells (U4.4) in response to viral infection. We infected U4.4 cells with CHIKV over a period of time of 72 h and investigated the global protein abundance at the early time point of infection, namely, 12 h post-infection (hpi) and at a later time point of infection, i.e., 60 hpi, using label-free quantitative mass spectrometry. First of all, we observed that U4.4 cells were persistently infected with CHIKV until this point, and minimal cytopathic effects were imparted to the cells. Mass spectrometry analysis followed by pathways analysis revealed that pathways such as metabolic processes, RNA metabolism, gene expression, and translation were regulated at these specific time points.

## 2. Materials and Methods

### 2.1. Cells, Virus, and Infection

African green monkey kidney-derived Vero cells (ATCC^®^ CCL-81™) were purchased from ATCC (Manassas, VA, USA). *Ae. albopictus* C6/36 cells were obtained from NCCS, Pune, India, and U4.4 cells were a kind gift from Prof. Robert Tesh from the University of Texas Medical Branch, Texas, USA. All cells were maintained in DMEM media (Himedia, cat. No. AL007A, Mumbai, India) supplemented with 10% fetal bovine serum (FBS) (Himedia, Cat. No. RM10409, Mumbai, India, and penicillin/streptomycin antibiotics. Culture medium for U4.4 cells was supplemented with 1X tryptose phosphate broth. All cells were maintained in a 5% CO_2_ incubator with humidity, and except for Vero cells (which were maintained at 37 °C), all cells were grown at 28 °C.

Cells were infected with a lab-adapted CHIKV clinical strain, IND-2010#01 (Accession no. JF950631.1) [32] The virus was grown alternatively between Vero and C6/36 cells, and for infection in U4.4 cells, CHIKV obtained from Vero cells were used after its quantification using plaque assays and further purification. For infection in U4.4, 1 × 10^6^ cells/well in 6-well plate or 4 × 10^6^ cells/T-25 flask were seeded, whereas in the case of Vero cells, wells with fully confluent monolayers were used. Cells were then added with sera-free DMEM media for 1 h and then incubated with CHIKV at a multiplicity of infection (MOI) 1 for 1.5 h. Cells were then washed with sera-free media to remove virus particles that had not entered the cell. and then 10% FBS-supplemented media was added to cells. Cells/media collected at this time were called 0 hpi (hour post-infection). Cells were then transferred to the incubator at 28 °C or 37 °C. Cells/media were collected at desired time points.

### 2.2. Cloning and Expression of CHIKV E1 Protein

RNA was isolated from CHIKV-infected Vero cells. The RNA was reverse transcribed using a PrimeScript one-step kit (Takara Bio Inc. Cat. RR055A, Shiga, Japan) with CHIKV E1 gene specific primers. Forward primer 5′-GCAGGTACCATGGTATTGGAGATGGAACTACTG-3′ and reverse primer 5′-GCAGGATCCTCGCACGACATGTCCGTTAAAG-3′ were made encompassing the region of the CHIKV E1 gene between the 27 and 302 amino acid region and cloned into the pET29a plasmid and transformed into competent *Escherichia coli* DH5α cells. The plasmid with the CHIKV E1 gene insert was transformed into competent *E. coli* BL21 (DE3) codon plus cells.

### 2.3. CHIKV E1 Protein Purification

The overnight culture of CHIKV E1 cloned *E. coli* was grown in terrific broth media at 37 °C until the OD reached around 1.0. It was induced at 18 °C with the final concentration of IPTG at 1 mM for 16 h and then centrifuged and lysed in the lysis buffer (Tris-Cl (pH 8.0) 50 mM, NaCl 150 mM, EDTA 2 mM, glycerol 5%, β-mercaptoethanol 2 mM, lysozyme, and PMSF 1 mM) followed by sonication. The suspension was centrifuged at 12,000× *g* for 30 min at 4 °C. The supernatant was then discarded, and 8 M urea was added to the pellet and incubated for 1 h (hour) at 37 °C with shaking. After the incubation, the urea suspension was centrifuged, and the supernatant obtained was mixed with Ni-NTA beads. The beads were eluted with Tris-Cl urea buffer containing 300 mM imidazole. The fractions were collected and stored at –80 °C. The urea was removed by dialysis membrane with Tris-Cl 50 mM (pH 8.0) and NaCl 300 mM for further use of protein.

### 2.4. SDS–PAGE, Staining, and Western Blotting

The SDS–PAGE and Western blot were done as per previous protocol [33]. Briefly, U4.4 cells were lysed in RIPA buffer (Tris-Cl 50 mM, pH 7.4, NaCl 150 mM, NP-40 1%, sodium deoxycholate 0.5%, SDS 0.1%) having a cocktail of protease inhibitors (Sigma, St. Louis, MO, USA, Cat. No. 11697498001) on ice for 30 min and then centrifuged at 10,000× *g* for 20 min. The clarified lysates were quantified by a Pierce™ BCA protein assay kit following the microplate assay protocol according to manufacturer’s protocol (Thermo Fisher Scientific, Waltham, MA, USA, Cat No. 23227), and 25 µg cell lysate of each sample was resolved on 12% SDS–PAGE gel (until the dye front came out from other end) and then proceeded for transfer onto nitrocellulose membrane (Bio-Rad, Hercules, CA, USA, Cat No. 1620112) for 1 h at 50 V in ice cold transfer buffer (25 mM Tris, 192 mM glycine, pH 8.3, 0.1% SDS and 20% methanol). The transfer quality was assessed by reversible staining the membrane with Ponceau S stain (Sigma, Cat. No. 41194, USA) and gel with Coomassie brilliant blue G-250 stain (50:40:10; methanol: water: glacial acetic acid *v*/*v* with 0.5% Coomassie brilliant blue G-250) followed by destaining in destaining solution (50:40:10; methanol: water: glacial acetic acid *v*/*v*). The membrane was blocked with 5% BSA and then probed with primary antibody (anti-His antibody 1:4000 (Santa Cruz, Cat. sc-8036-HRP, Dallas, TX, USA) or anti-CHIKV E1 mice sera 1:3000 in PBS + 2.5% BSA). This was followed by washing with 0.1% Tween-20 in PBS (PBST) solution thrice for 10 min each. The E1 antisera-probed membrane was then incubated with anti-mice HRP antibody (1:6000 dilution) (Novus Biologicals, Cat. NB7539, Centennial, CO, USA) in PBST + 2.5% BSA for 1 h at RT, followed by washing with PBST for 3 × 10 min and then visualized using SuperSignal^®^ West Pico Chemiluminescent Substrate (Thermo Fisher Scientific Inc., Waltham, MA, USA) in the Bio-rad ChemiDoc MP system (Hercules, CA, USA). The image contrast and brightness were adjusted equally across the whole images for best visualization of protein bands. The uncropped images of blots used in the study as well as of the validation of antibody specificity are included in supplementary information in Figures S3 and S4.

### 2.5. Anti-CHIKV E1 Antibody Generation

The purified recombinant CHIKV E1 protein was injected into four female Balb/c mice (4–6 weeks) with FCA (Freund’s complete adjuvant) at a 100 µg/mice concentration. The booster doses of the protein (50 µg/mice) were given at regular intervals. The blood was collected at regular intervals, and sera were isolated from whole blood and transferred to a fresh tube. The sera were then added with sterile sodium azide at a concentration of 0.002%, aliquoted, and then kept at −80 °C (for future use).

### 2.6. RNA Isolation and Real-Time PCR

RNA isolation and real-time PCR were done as per previous protocol [33]. Briefly, total RNA was isolated using TRIzol (Thermo Fisher Scientific Inc., Waltham, MA, USA). RNA was dissolved in DEPC-treated water, and RNA was quantified. One-step SYBR green real-time PCR was carried out on a PIKOREAL 96 real-time PCR system (Thermo Fisher Scientific Inc., Waltham, MA, USA). A total of 300 ng total RNA per reaction was used with 0.3 μM of each primer with QuantiTect PCR kit (Qiagen, Hilden, Germany). The RT-PCR conditions for the one-step RT-PCR consisted of a 30 min reverse transcription step at 50 °C and then 2 min of initial denaturation at 95 °C, followed by 40 cycles of PCR at 95 °C without holding time (denaturation), 60 °C for 30 s (annealing), and 72 °C for 30 s (extension). Small subunit ribosomal protein 7 (RPS7) was used as an internal control. Primer details are included in Supplementary Table S1.

### 2.7. Plaque Assay

A plaque assay was done following our previous protocol [34]. Briefly, culture medium was replaced with sera-free for 1 h. The supernatants of time point samples of U4.4 cells were initially diluted at 1:10 and added to the first well and then was diluted at 1:2 in the rest of the wells. Each sample was titrated in triplicate. The virus was allowed to bind for 1.5 h, and then the media was changed. The wells were then added with 1% carboxymethyl cellulose (CMC) (Cat. C4888, Sigma Aldrich, St. Louis, MO, USA), and plates were transferred back to the incubator at 37 °C for 72 h. The cells were fixed with 4% paraformaldehyde for 1 h. After the fixing, cells were added with the crystal violet stain (0.25%) for 30 min. The stain solution was discarded, and wells were rinsed with tap water. The plaques were calculated as plaque-forming units (pfu) = (number of plaques)/(dilution × volume of the virus).

### 2.8. Immunofluorescence Assay

Vero cells were cultured in DMEM complete media in 6-well plates containing sterile glass coverslips. The cells were infected with chikungunya virus at MOI 1 for different time points. The cells were then fixed with 4% paraformaldehyde for 30 min and permeabilized with PBST (PBS + 0.1% triton-X-100) for 30 min. Cells were then blocked with PBS + 5% BSA for 1 h and then incubated with anti-CHIKV E1 mice sera at 1:200 dilution in PBS + 2.5% BSA overnight. The following day, washing was done using PBST (PBS + 0.1% Tween-20) thrice for 10 min. The cells were then added with secondary antibody (anti-mice Alexa 488) at 1:400 dilution in PBS + 2.5% BSA (PBS + 0.1% Tween-20), followed by washing with PBS + 0.1% Tween-20 for 3 × 10 min. The cells were immersed in DAPI for a short interval and then visualized with a confocal microscope (Eclipse Ti2, Nikon corporation, Minato, Tokyo, Japan).

### 2.9. Cell Lysis and Sample Preparation for Mass Spectrometry

For mass spectrometric analysis of the U4.4 cell global proteome, cells were seeded in 9 T-25 flasks (4 × 10^6^ cells/flask) and allowed to grow at 28 °C with 5% CO_2_. Upon reaching 70% confluency, the cells were infected with CHIKV at MOI 1, following an earlier protocol. The triplicate samples of uninfected cells (Control 1, Control 2, and Control 3), 12 hpi cells (Sample 12H 1, Sample 12H 2, and Sample 12H 3), and 60 hpi cells (Sample 60H 1, Sample 60H 2, and Sample 60H 3) were washed twice with ice-cold PBS. Cells were scraped using a cell scraper and collected in tubes. Cells were centrifuged at 200× *g* at 4 °C. The cells were then lysed with 1 mL lysis buffer/flask for 15 min in ice. The lysate was centrifuged at 10,000× *g* at 4 °C for 15 min. The supernatant was transferred to a fresh vial and was quantified using a Pierce™ BCA protein assay kit (Thermo Fisher Scientific, Cat No. 23227, USA). Then 25 µg protein from each sample was reduced with 5 mM TCEP and further alkylated with 50 mM Iodoacetamide and then digested with trypsin (1:50, trypsin/lysate ratio) for 16 h at 37 °C. Digests were cleaned using a C18 silica cartridge to remove the salt and dried using a speed vac (Thermo Fisher Scientific Inc., Waltham, MA, USA). The dried pellet was suspended in buffer A (5% acetonitrile, 0.1% formic acid).

### 2.10. Mass Spectrometric Analysis of Peptide Mixtures

Experiments were performed on an Ultimate 3000 RSLC nanosystem coupled with an Orbitrap Eclipse (Thermo Fisher Scientific Inc., Waltham, MA, USA). Then 500 ng was loaded on a C18 column 50 cm, 3.0 μm Easy-spray column (Thermo Fisher Scientific Inc., Waltham, MA, USA). Peptides were eluted with a 0–40% gradient of buffer B (80% acetonitrile, 0.1% formic acid) at a flow rate of 300 mL/min and injected for MS analysis. LC gradients were run for 100 min. MS1 spectra were acquired in the Orbitrap (R = 240 k; AGC target = 400,000; Max IT = 50 ms; RF Lens = 30%; mass range = 400–2000; centroid data). Dynamic exclusion was employed for 10 s, excluding all charge states for a given precursor. MS2 spectra were collected in either the linear ion trap (rate = turbo; AGC target = 20,000; MaxIT = 50 ms; NCEHCD = 35%).

### 2.11. Data Processing

All samples were processed, and the RAW files generated were analyzed with Proteome Discoverer (v2.4) (Thermo Fisher Scientific Inc., Waltham, MA, USA) against the customized database of *Aedes albopictus* Foshan (downloaded in April 2021) from Vectorbase merged with CHIKV proteome. For the Sequest search, the precursor and fragment mass tolerances were set at 10 ppm and 0.5 Da, respectively. The protease used to generate peptides, i.e., enzyme specificity, was set for trypsin/P (cleavage at the C terminus of “K/R: unless followed by “P”) along with a maximum missed cleavages value of two. Carbamidomethyl on cysteine as fixed modification and oxidation of methionine and N-terminal acetylation were considered as variable modifications for the database search. Both peptide spectrum match and protein false discovery rate were set to 0.01 FDR.

### 2.12. Protein Abundance Analysis

Protein abundance status of all samples in comparison to uninfected was used to infer relative abundance (Label-Free Quantitation) of specific proteins during the infected state at the different time points. Label Free Quantification was performed using the minora mode of Proteome Discoverer software (Thermo Fisher Scientific Inc., Waltham, MA, USA). In the peptide and protein quantifier node, the “Top N peptides used for quantification” parameter was set to 3. Perseus software (Max Planck Institute of Biochemistry, Martinsried, Germany) was used for differential protein analysis and statistical testing. Protein abundance values were filtered based on valid values (at least present in 8 samples) among all the quantified proteins. Filtered values were Log2 transformed, followed by default Z-score normalization settings available in Perseus. ANOVA testing was used to identify significant proteins among the experimental conditions. Statistical significance was considered for *p* values less than or equal to 0.05. Z score normalization-derived abundance values of the significant proteins were then used for bioinformatics data visualization using In-house R Programming Scripts. The reproducibility of biological replicates was accessed by principal component analysis. The proteins that were differentially abundant by either Log2 fold change of +0.58 or ≤−0.58 as compared to uninfected were included in the further analysis. The functional enrichment was performed using open source ShinyGO v0.66 (ShinyGO 0.76 (http://bioinformatics.sdstate.edu/go76/, accessed on 31 March 2022)). The *p*-value cut-off (FDR) was set to 0.05. The gene details corresponding to the IDs were fetched from the Uniprot database.

### 2.13. Orthologue Analysis Using Aedes aegypti Genome

Proteins IDs of *Ae. aegypti* were converted into *Ae. albopictus* ID using g: Profiler toolkit [35]. A hybrid dataset was created using both *Ae. aegypti* and *Ae. albopictus* proteomes from the VectorBase. The raw proteome dataset of *Ae. albopictus* obtained from the present study was annotated using this hybrid database. The clusterProfiler R package was used for enrichment and pathway analysis of all orthologous proteins between *Ae. aegypti* and *Ae. albopictus.* Significantly expressed proteins were compiled on the basis of early and late time points of CHIKV infection and were taken for downstream analysis.

### 2.14. Statistical Analysis and Software

Statistical analyses for plaque assay analysis and real-time PCR were performed using one-way ANOVA. The significance level was determined at *p* < 0.01 (**), *p* < 0.001 (***), and *p* < 0.0001 (****). The analyses were done using GraphPad prism software (version 9.1.1, GraphPad Software, San Diego, CA, USA). The pie chart was prepared using Microsoft Excel (Microsoft corporation, Redmond, Washington, DC, USA).

## 3. Results

### 3.1. Replication Kinetics of CHIKV in Ae. albopictus Cells

**Figure 1:**
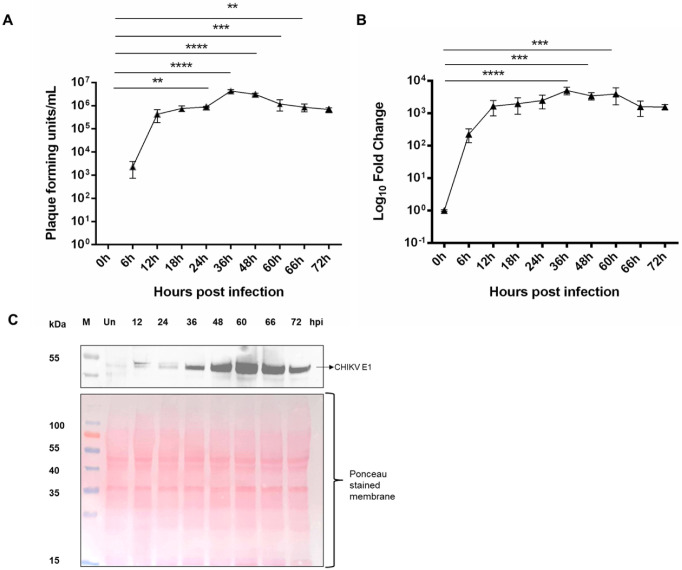
CHIKV kinetics in U4.4 cells: (**A**) plaque assay of different time points of CHIKV infection in U4.4 cells at MOI 1, error bars represent standard deviation from three biological repeats and technical repeats; (**B**) quantitative PCR of CHIKV E1 gene at different time points of infection in U4.4 cells; error bars represent standard deviation from three repeats from three biological repeats and technical repeats; and (**C**) Western blot expression profiling of different infection time points of CHIKV E1 protein in CHIKV-infected U4.4 cells at MOI 1 (top image). Nitrocellulose membrane stained with Ponceau S immediately after Western transfer used for CHIKV E1 detection (bottom image). *p* < 0.01 (**), *p* < 0.001 (***), and *p* < 0.0001 (****). The uncropped blot images are included in the supplementary information.

The growth kinetic analysis of CHIKV in U4.4 was done at different time points in U4.4 cells at MOI 1. We found that the virus replication peaked at 24 to 36 hpi and then reduced slightly till 72 hpi, as observed through the plaque assay and quantitative PCR. The PFU/µL was 4.3 × 10^5^, 8.8 × 10^5^, 4.3 × 10^6^, 3.03 × 10^6^, 1.2 × 10^6^, 8.6 × 10^5^, and 5.1 × 10^5^ at 12 hpi, 24 hpi, 36 hpi, 48 hpi, 60 hpi, 66 hpi, and 72 hpi, respectively (Figure 1A), indicating around 2-, 10-, and 7-, and 2.7-, 2.0-, and 1.2-fold change at 24 hpi, 36 hpi, and 48 hpi, and 60 hpi, 66 hpi and 72 hpi w.r.t. 12 hpi. CHIKV E1 gene (primer sequence in Table S1) expression followed a similar pattern to the plaque assay (Figure 1B). Further, the replication was quantified by estimation of CHIKV E1 protein during infection using in-house mice-raised anti-CHIKV E1 sera (Figure S1). From 0 hpi to 72 hpi, it was observed that there was an increase of the virus from 12 hpi to 48 hpi and a slight reduction afterward at 60 hpi, which was again followed by an increase at later time points (Figure 1C).

### 3.2. Global Proteomics Analysis of Aedes Proteins during CHIKV Infection

We then proceeded to determine the impact of viral infection on the proteome of U4.4 cells. The CHIKV kinetics data in U4.4 cells (Figure 1A–C) showed that virus growth was significant as early as 12 hpi. Therefore, to study changes in the proteome early during infection, the 12 hpi time point was selected. Further, we also observed a peak in virus growth at 36 to 48 hpi at an MOI of 1 followed by a plateau at 60 hpi. To study the pathways that might have a role in controlling infection, 60 hpi was chosen, as we believe that at this time point, pathways involved in regulating virus growth would be active, thereby effectively controlling the viral growth, as was evident from the active virus replication results provided above.

**Figure 2:**
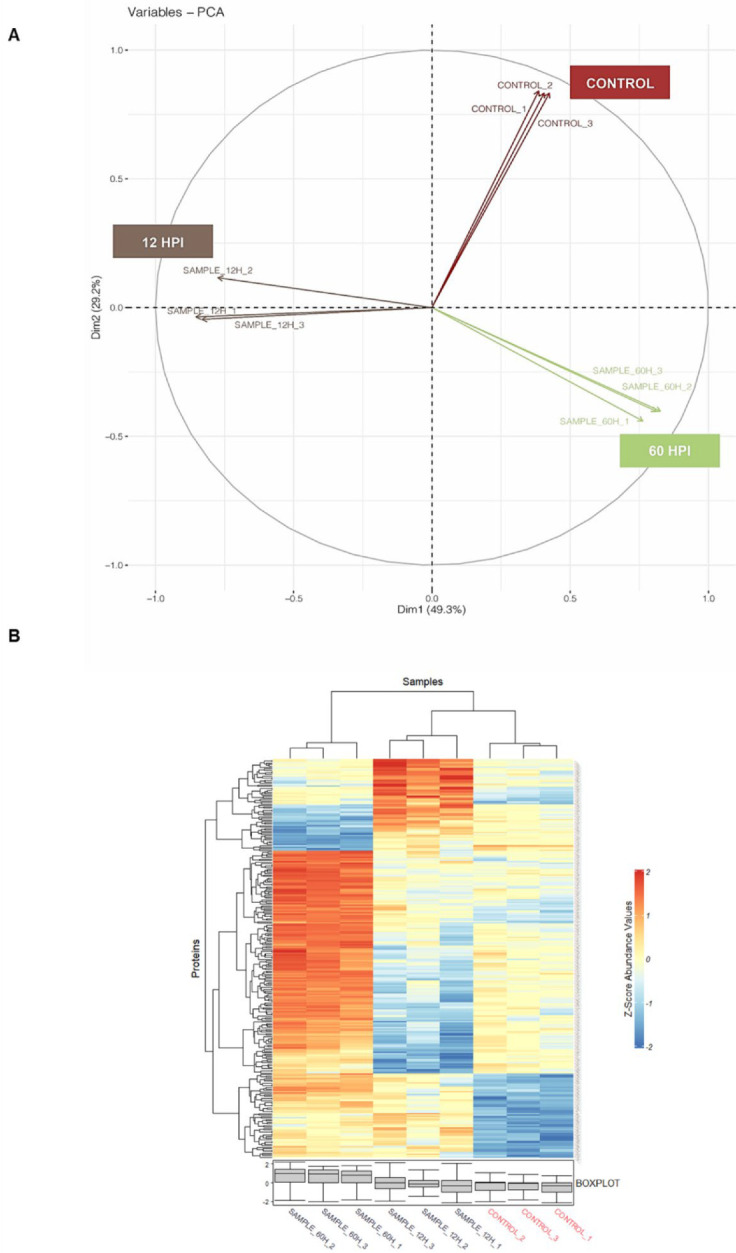
Mass-spectrometric analysis of U4.4 cell samples for global proteome analysis upon CHIKV infection: (**A**) principal component analysis (PCA) of triplicate samples of uninfected (control), 12 hpi, and 60 hpi of U4.4 cell lysate, as shown by different colour for each time-point; (**B**) Z-score analysis of triplicate samples of uninfected (control), 12 hpi, and 60 hpi CHIKV-infected U4.4 cell lysate. The Z-score value ranges from −2 to 2, and the blue color indicates negative values, and the red color indicates positive values of Z-scores. The box plot shows the locality, spread, and skewness of Z-score values of each of the replicates.

To evaluate the impact of CHIKV infection on early and late time points of infection, we employed label-free mass spectrometry analysis of U4.4 cells as uninfected, 12 hpi, and 60 hpi upon CHIKV infection at MOI 1. Mass spectrometric analysis of uninfected and CHIKV-infected U4.4 cells revealed the presence of a total of 5280 proteins in all time points, in at least 8 of 9 replicates. The raw data are provided in Supplementary information S1 and S1a. Principal component analysis (PCA) of the uninfected samples (control), 12 hpi, and 60 hpi (in triplicate) revealed that the samples were positively correlated (Figure 2A). This was also shown by correlation analysis (Figure S2). The abundance variation between triplicates of three time points is shown in the heatmap in Figure 2B, indicating that triplicate samples of each time point were correlated with abundance level.

The *Ae. albopictus* proteome was compared to the *Ae. aegypti* dataset for functional annotation. The extensive research on *Ae. aegypti* led to a very well-annotated proteome as compared to *Ae. albopictus*. The resulting analysis presented a total of 3145 proteins that were found to be common and were taken for further analysis. Pathway analysis revealed that the organonitrogen compound metabolic process, macromolecule metabolic process, biological regulation, regulation of cellular process, and protein metabolic process were among the major ones to be regulated, and several of these were also time point dependent (Supplementary information S2).

The statistically significant proteins (*p*-value < 0.05) were selected for further analysis, such as the difference in abundance when compared to uninfected, their difference in abundance between time points, and pathway analysis of these regulated proteins. The differential abundance analysis of the global proteome of CHIKV-infected U4.4 cells revealed that at 12 hpi, 195 proteins had significant abundance with log2 fold change (log2 FC) of ≥ +0.58 (*p*-value < 0.05), and 134 proteins had reduced abundance with log2 FC ≤ −0.58 (*p*-value < 0.05), whereas at 60 hpi, 907 proteins showed higher abundance, and 413 proteins had reduced abundance (Supplementary information S3). The top 30 proteins from 12 hpi and 60 hpi that were most affected, shown in Supplementary information S1, indicate that diverse types of proteins were modulated, and many of them were uncharacterized.

**Figure 3:**
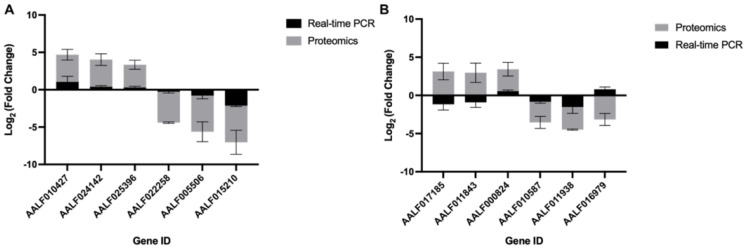
Quantitative PCR and mass spectrometric analysis of top differentially abundant proteins: (**A**) Log2 Fold change abundance analysis of mass spectrometric data of selected set of genes as well as their transcript expression profile, determined by real-time PCR analysis at 12 hpi, and (**B**) 60 hpi in U4.4 cells upon CHIKV infection. The proteins whose abundance was altered most in the analysis of mass spectrometric data were selected, and their transcript levels were compared to 0 hpi with real-time PCR. Error bars represent standard deviation (sd). The experiments were done in triplicate.

To ascertain if the proteins’ abundance modulation in *Aedes* cells were at a translational or transcriptional level, we estimated the transcript expression profiling of the top three whose abundance was changed the most as compared to uninfected, namely, at 12 hpi, (AAFL010427, AALF024142, AALF025396, AALF015210, AALF005506, and AALF022258) and at 60 hpi (AALF0171858, AALF011543, AALF00824, AALF010587, AALF011938, and AALF016979). The real-time PCR analysis was done with RNA isolated from 0 hpi, 12 hpi, and 60 hpi CHIKV-infected U4.4 cells and with primers specific (Table S1) to the genes given above. The expression profiling of the transcripts at 12 hpi as compared to 0 hpi revealed that their expression followed a similar pattern of modulation to that of their protein abundance; albeit, the extent of modulation at the level of transcription was low. However, in the case of 60 hpi, the pattern of transcript expression did not follow the protein abundance pattern in each case (Figure 3).

### 3.3. Pathway Analysis of Aedes Proteins during CHIKV Infection

**Figure 4:**
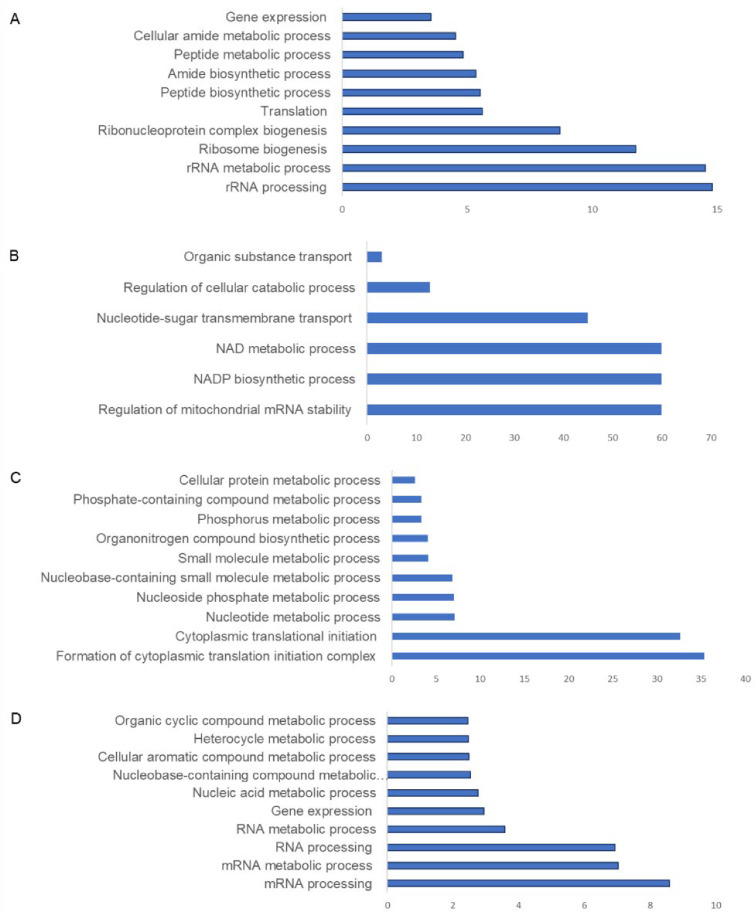
Pathway analysis of CHIKV-infected U4.4 cells in the pie chart: (**A**) negatively regulated pathways at 12 hpi in comparison to uninfected cells; (**B**) abundant pathways at 12 hpi in comparison to uninfected cells; (**C**) negatively regulated pathways at 60 hpi in comparison to uninfected cells; and (**D**) abundant pathways at 60 hpi in comparison to uninfected cells. *X*-axis depicts fold enrichment for pathways, and *Y*-axis indicates pathways.

**Figure 5:**
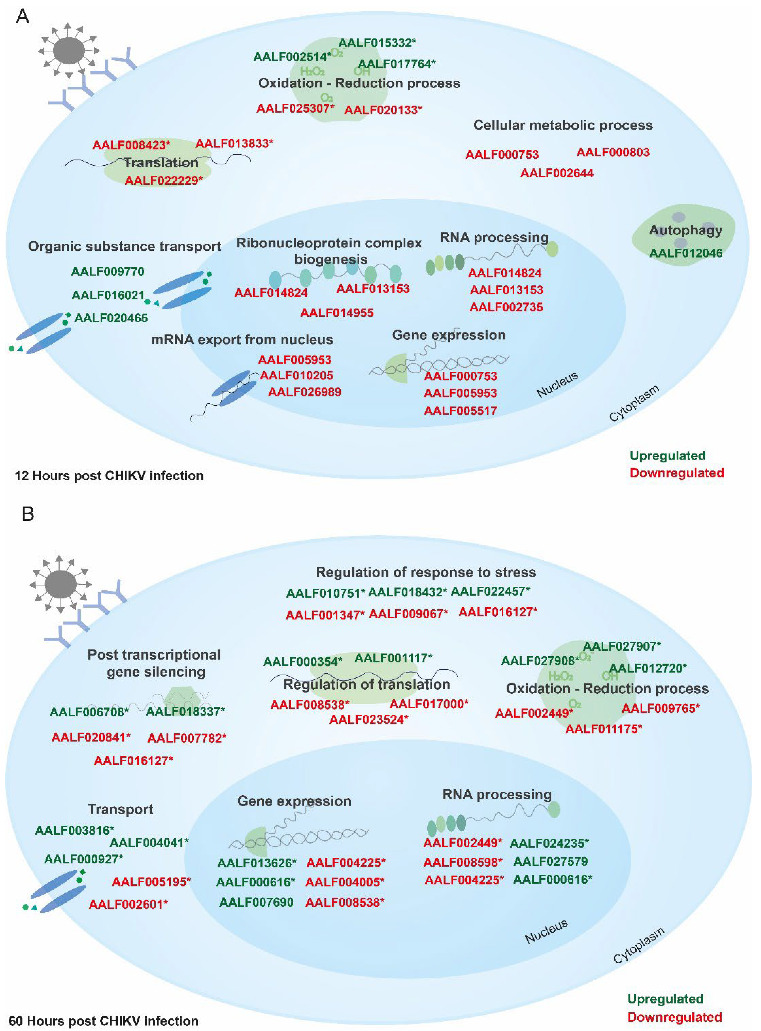
Major pathways affected during (**A**) early (12 hpi) CHIKV infection in U4.4 cells. During the initial time period of infection, viruses are known to halt host translation, leading to negative impacts on other pathways. Few of these decreased pathways included translation, RNA processing, and the nitrogen compound metabolic process, whereas pathways such as transport, and regulation of autophagy were abundant as compared to uninfected; and (**B**) late (60 hpi) CHIKV infection in U4.4 cells. At the late phase of infection, pathways such as translation, the nucleotide metabolic process, and the primary metabolic process were negatively impacted, while pathways such as RNA processing, ribonucleoprotein complex biogenesis, and ncRNA processing were increased in abundance. A few of the representative proteins are also shown in green (upregulated) and red (downregulated). Asterisk (*) represents proteins taken from re-annotation analysis.

CHIKV infection was found to modulate the cellular proteome both during early and later time points post-infection. Pathway analysis of significantly abundant proteins revealed that at 12 hpi, proteins belonging to pathways such as rRNA processing, rRNA metabolic processes, ribonucleoprotein complex biogenesis, translation, and gene expression were adversely impacted in terms of abundance (Figure 4A and Figure 5A and Supplementary information S2 and S3), whereas enriched pathways included regulation of mitochondrial RNA stability, organic substance transport, NADP biosynthetic processes, the oxidation–reduction pathway, and nucleotide sugar transmembrane transport, whose abundance was increased (Figure 4B and Figure 5A and full list in Supplementary information S2 and S3).

At 60 hpi, negatively impacted pathways included posttranscriptional gene silencing, translation, the organonitrogen compound metabolic process, among others (Figure 4D and Figure 5B and Supplementary information S2 and S3), whereas enriched pathways included ribonucleoprotein complex biogenesis, the regulation of immune effector process, positive regulation of response to stimuli, ubiquitin-dependent protein catabolic process ncRNA processing, and transcription (Figure 4C and Figure 5B and full list in Supplementary information S2 and S3).

Further, a comparative analysis of proteins modulated from 12 hpi to 60 hpi was done. It was found that 152 proteins showed reduced abundance at 60 hpi in comparison to 12 hpi, largely associated with pathways such as metabolic processes, macromolecule modification, protein ubiquitination, and phosphorous metabolic processes. The analysis revealed that 510 genes had high abundance at 60 hpi as compared to 12 hpi belonging to pathways such as RNA metabolic processes, nucleic acid templated transcription, gene expression, etc. (Supplementary information S3).

## 4. Discussion

Viruses infect the host cell and manipulate the host’s cellular machinery to support the replication of its genome [36]. During infection, the host proteins play varied roles; some help in viral genome replication, translation, and protein processing, while other host proteins participate in thwarting the establishment of the virus within the cell [37]. Additionally, virus infections induce oxidative stress in the host cells, which promotes viral RNA capping and genome replication [38] but in turn negatively affects the host proteome due to inhibition of host cell translation [39]. In those cells where the virus needs to survive for long periods without affecting the host cell metabolism, the interactions between the host and virus are tightly orchestrated to allow survival of both the virus and the host [40,41]. These have been reported in several persistent human pathogenic viruses such as HCV [42] and herpes [43]. Most arboviruses are persistent in their vectors and are known to utilize several mechanisms to ensure their continued survival in the vector [44,45].

The present study was undertaken to evaluate the nuances of survival of CHIKV in *Ae. albopictus* cells and provide a birds-eye view of global proteome changes that occur in the vector during the initial and later time points of viral infection. The cells were able to tolerate the virus to the late infection time points, indicating that the virus is persistently replicating inside the *Aedes* cells, corroborating earlier reports involving other arboviruses such as Sindbis virus, Culex Y virus, and West Nile virus [44,45,46]. Viruses are known to induce oxidative stress in host cells, which can have both pro-viral and antiviral impacts on the cells [38,47,48]. Either way, this oxidative stress leads to activation of pathways such as apoptosis, as well as downregulation of pathways such as translation, and RNA processing, which is often associated with the formation of RNP complexes called stress granules [49]. It should however be noted that during stress, alternate crucial stress response pathways are kick-started to promote cell survival. Stress granules are known to be induced early in CHIKV infection, although later on, non-structural protein 3 (nsP3) has been reported to hinder their assembly [50,51]. It was also visible from the study, as the number of genes in which the whole abundance was enriched were increased at a later time point. The present study is one of the few on *Aedes* that brings out the relevance of the pathways pertaining to translation and gene expression during CHIKV infection, with these pathways negatively impacted during early time points and eventually getting enriched during the later time point. At 60 hpi, we observed intracellular viral replication reaching its plateau, and cells became persistently infected with the virus, indicating that immune pathways of cells are maintaining virus numbers at check. This was consistent with the previous report in U4.4 cells, where cells were persistently infected with Semliki forest virus (SFV) [52].

Infection of viruses leads to activation of immune pathways such as the RNAi pathway and antioxidant pathway and continue to be active even during persistence of infection; however, various defense mechanisms are activated at the later time point of infection to counter the virus as well as virus-induced oxidative stress [53,54,55]. Additionally, we found that the abundance level of proteins such as heme oxygenase (AALF009424) and poly ADP ribose polymerase (AALF018432) was increased. These are known to exert an antiviral effect [56,57]. We believe that during the late time point of infection, mosquito cells activate the immune system to avoid cell death as well as to control viral growth to favor their survival.

Mass spectrometry studies on *Ae. aegypti* or Dicer inactive *Ae. albopictus* cells or whole mosquitoes upon CHIKV infection from the past have highlighted the roles of different pathways during infection [23,58,59]. These include translation, oxidation–reduction, phosphate-containing compound metabolic process, and ribosome biogenesis, among the major ones, indicating that these pathways are affected by virus infection and are helping somehow in the survival of the host cells and favor viral replication. Additionally, various bottom-up studies have analyzed the impact of virus on *Aedes* cells in terms of the global canonical proteome. These studies provide information about the canonical proteins; however, these studies are limited in their ability to identify the specific isoforms of proteins that might be playing functional roles, including splice variants, PTMs, and amino acid variants. Nevertheless, the present study is a straightforward analysis providing overall information on proteins that play roles in metabolism, survival, growth, etc., that are making possible the co-survival of both viruses and vectors, and it may guide the future work on individual proteins/pathways as well as their proteoforms to determine their roles in infection.

## 5. Significance of the Present Study

This study is the first to evaluate the proteome of the Dicer active *Ae. albopictus* cell line during early and late time points of arbovirus infection. One of the critical issues that we encountered during data analysis was the poor annotation of the *Aedes* proteome database, thereby impeding in-depth analysis of protein complexity per se in *Aedes*, especially during an arboviral infection. Owing to the experimental design, analysis of the proteoforms that might play important roles in infection and in *Aedes* physiology could not be performed. We believe that top-down approaches in combination with bottom-up shotgun proteomics approaches will yield a plethora of information regarding *Aedes* physiology during pathogen infection. Additionally, there is a dearth of extensive insect-specific databases, and the present study will contribute to narrowing that knowledge gap.

In conclusion, this study found that CHIKV persistently infects the Dicer active *Ae. albopictus* U4.4 cells. The pathway analysis of statistically significant and differentially expressed proteins revealed that during early phases of virus infection, major cellular processes such as translation, gene expression, RNA processing (rRNA and ncRNA), cellular metabolic processes, and ribosome biogenesis were negatively affected. The global inhibition of translation could be due to virus-induced oxidative stress in cells, which is known to lead to cytoplasmic accumulation of stalled preinitiation translation complexes due to phosphorylation of eIF2a by cellular kinases. Proteins involved in the transport of organic molecules, as well as oxidation–reduction, were upregulated. At late stages of infection, more proteins were upregulated than downregulated. These belonged to pathways such as gene expression, ribosome biogenesis, and others, including those involved in metabolic processes such as nitrogen compounds, cyclic compounds, aromatic compounds, and nucleic acids. We believe that cells modulate their gene expression in response to infection by virus to promote their survival. Overall, this global mass spectrometry study of *Aedes* cells upon CHIKV infection enrich the data about the currently available studies in *Aedes* mosquitoes or cells.

## Data Availability

The mass spectrometry proteomics data have been deposited to the ProteomeXchange Consortium via the PRIDE partner repository [60,61] with the dataset identifier PXD033252.

## Supplementary Materials

The following supporting information can be downloaded at: https://www.mdpi.com/article/10.3390/proteomes10040038/s1. Supplementary information S1: Mass spectrometry data of CHIKV-infected U4.4 cells; Supplementary information S1a: Peptide CHIKV-infected U4.4 cells; Supplementary information S2: Orthologue analysis of Ae. aegypti with Ae. Albopictus; Supplementary information S3: Pathway analysis of upregulated and downregulated proteins; Supplementary Table S1: List of primers used in real-time PCR and Supplementary figures: Figure S1: Cloning, expression, and antibody generation of CHIKV E1, Figure S2: Comparison of global abundance of Aedes proteins among triplicates, Figure S3: Validation of antibodies used in western blots, and Figure S4: Raw figures of gels and western blots used in the manuscript.
