# Supplementary material for: Impact of CHIKV Replication on the Global Proteome of Aedes albopictus Cells
Source: Proteomes. 2022 Nov 10;10(4):38. doi: 10.3390/proteomes10040038 (PMC9680348; doi:10.3390/proteomes10040038)
Supplement: Supplementary file 1 [file proteomes-10-00038-s001.zip › proteomes-1869526-supplementary/Supplementary Material/Supplementary Table S1 and Figures S1-S4.pdf]

Table S1: List of primers used in real-time PCR

	Gene ID	Forward Primer	Reverse Primer
1	AALF011843	CGTTCTTGAGTCGGGAAGAAA	CTTCTGGCAGATGTCAGCTATT
2	AALF016979	CTACAGCGCGTCACCTATTT	CACGTAGCACTCCACATCAA
3	AALF011938	GCACTGGTGGGTATGACTTTAG	CAGAAGCGAACTTCCTCAT
4	AALF017185	GGAAAGCAACGATCCAAGAAG	TTACGCAGTTTGGTGGTTATTT
5	AALF000824	ACGGAACTGACCAGGAAATG	CTGGCAGGTGTATTCGACTT
6	AALF011843	CGTCGACCATGAGGAATCTAAG	TGCGCATTCAGCATCAAATAC
7	AALF10427	CCAGTGGCATTGAGTGAGAA	CCAGCATTACATCCCTCCTTATC
8	AALF024142	ACGCCACTATGCCAACAA	GTAGCCGAAGAATCCGATCAA
9	AALF025396	GGAACCTACCGTCAGTTGTT	TTCCTTTCCGATGGTGTAGTG
10	AALF015210	GATGTCCTCGGGAAACGATTAG	CCTTCATCCAGTCCTTGAACAG
11	AALF005506	ACGAAGCGTACGAGAAGATTG	CGGCTGTTCCCTGTGTATTAG
12	AALF022258	AATGTCTCGCTATCCACACG	CGGGCCGTAGTAAGAGAATG
13	AALF004605	CTTCGATATGCGTGTGTTCAAG	CTCACAGACACATCCAACCA
14	AALF005111	AAGATCCGTCGCAGTTCTATG	GTGGCAAATCGGTCTTGTAATG
15	AALF009424	AGCTGGCTTTCGCTCTTTAT	TGTTGCGTCCGATGGTATTC
16	AALF001602	AAGCTGAAGGGTGACGATATG	GTCACAAACACGCTCCAAAC
17	AALF023524	GCCCACGATAACAACCTCAA	GTTTCTCCCAGAACTGCCTATC
18	AALF019400	GGGTGGTTCCAACTTCATCA	CAGACCGTAGGTCACCAAATAC
19	AALF008592	TGGACTCGGATTCAGATGAAAG	CATCGTCTCCGTCTTCCTTATC
20	AALF008541	GAGACACTTCGCGGATCAA	GCTCCTCCATAGCGACAAAT
21	AALF005515	CTAAGCAGTGCCGGGTTATT	TTCTTCACCTCCACCCTGTA
22	RPS7	GCAAGAAGGCTATCGTGATCTA	CGGAGAACTTCTTCTCCAACTC
23	CHIKV E1	TACCCATTTATGTGGGGC	GCCTTTGTACACCACGATT

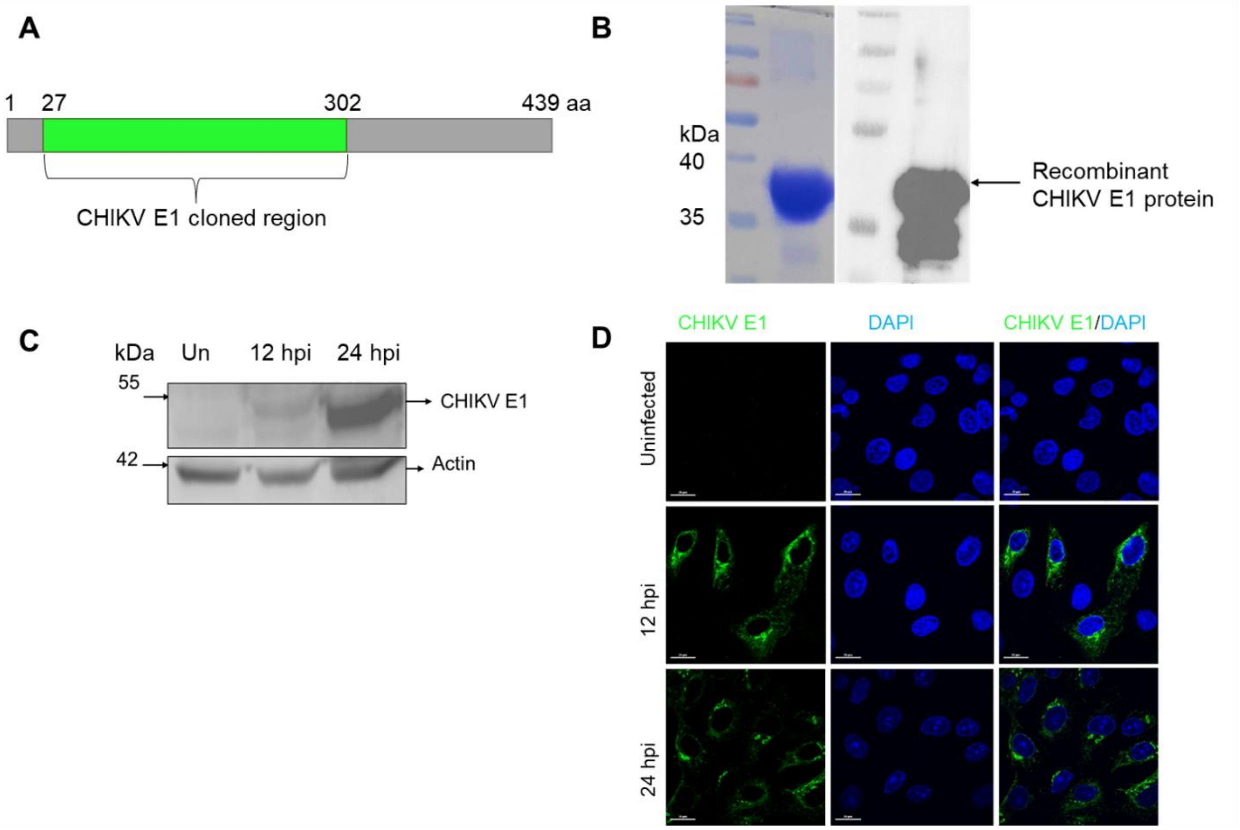

Figure S1. Cloning, expression, and antibody generation of CHIKV E1. A) The internal region of CHIKV E1 (highlighted by green colour) was amplified using gene-specific primers from CHIKV infected total RNA; B) The affinity chromatography purified recombinant CHIKV E1 protein was resolved on 12 % SDS PAGE and stained with Coomassie brilliant blue as well as same was immunoblotted with anti-His antibody and showed intense band at size range of 37 kDa; C) Western blot of uninfected and CHIKV infected Vero cells at MOI 1. The nitrocellulose membrane was incubated with anti-CHIKV E1 mice sera at 1:3000 dilution, and after washing, the membrane was probed with anti-mice HRP antibody at 1:6000 dilution. Actin antibody was used as a loading control at 1:4000 dilution, and D) Immunofluorescence assay of uninfected and CHIKV infected Vero cells at different time points at MOI 0.1. The cells were infected, permeabilized and blocked as mention in the protocol. After primary antibody (anti-CHIKV E1 sera) incubation, secondary antibody (anti-mice Alexa 488 antibody) and DAPI incubation, cells were visualized in confocal microscope.

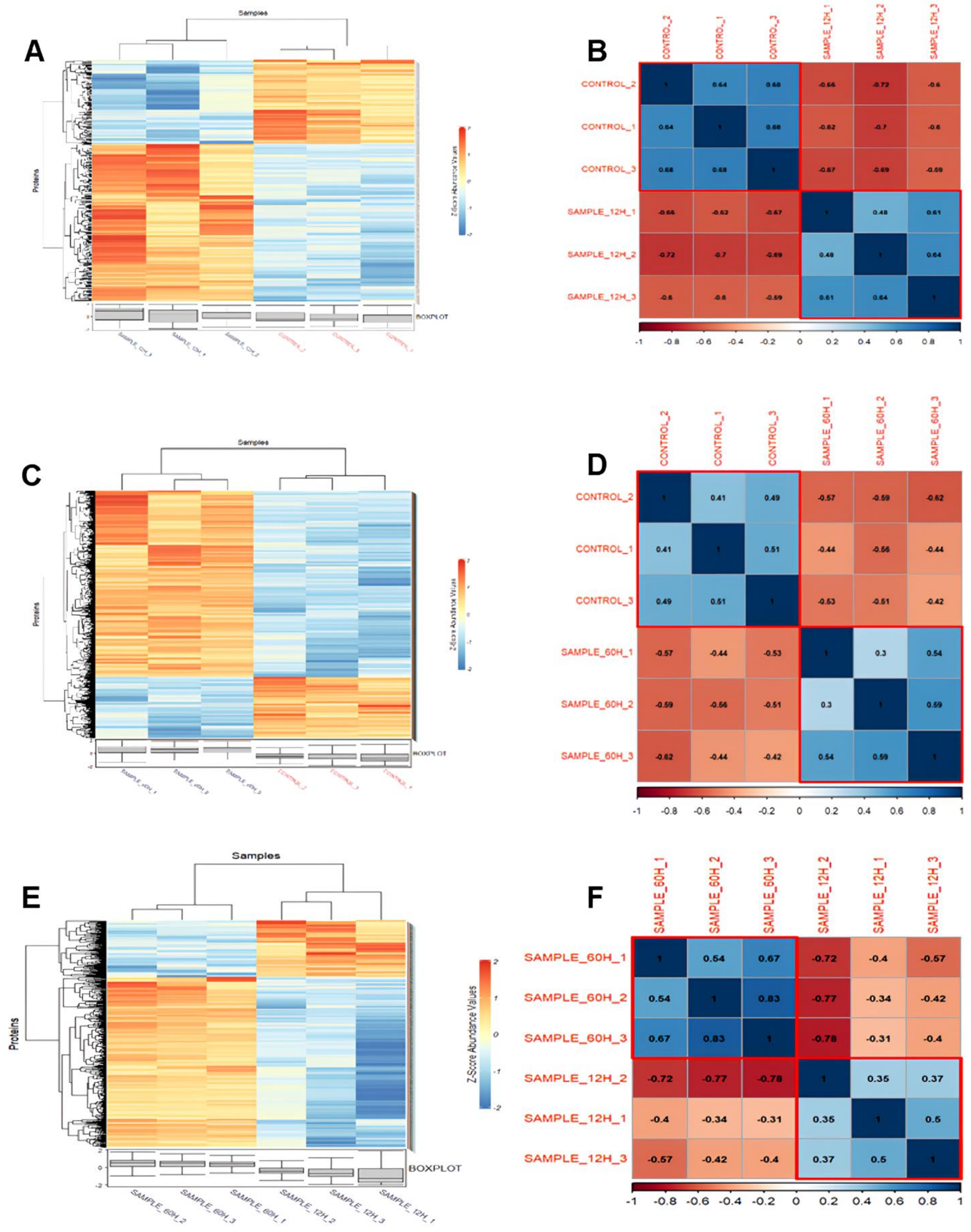

Figure S2. Comparison of global abundance of Aedes proteins among triplicates. A) Heat map of global comparison of 12 hpi triplicates with control triplicates (uninfected); B) Correlation

coefficient analysis of 12 hpi and control sample; C) Heat map of global comparison of 60 hpi triplicates with control triplicates (uninfected); and D) Correlation coefficient analysis of 60 hpi and control sample; E) Heat map of global comparison of 60 hpi triplicate samples with 12 hpi triplicate samples, and F) Correlation coefficient analysis of 60 hpi and 12 hpi triplicate samples.

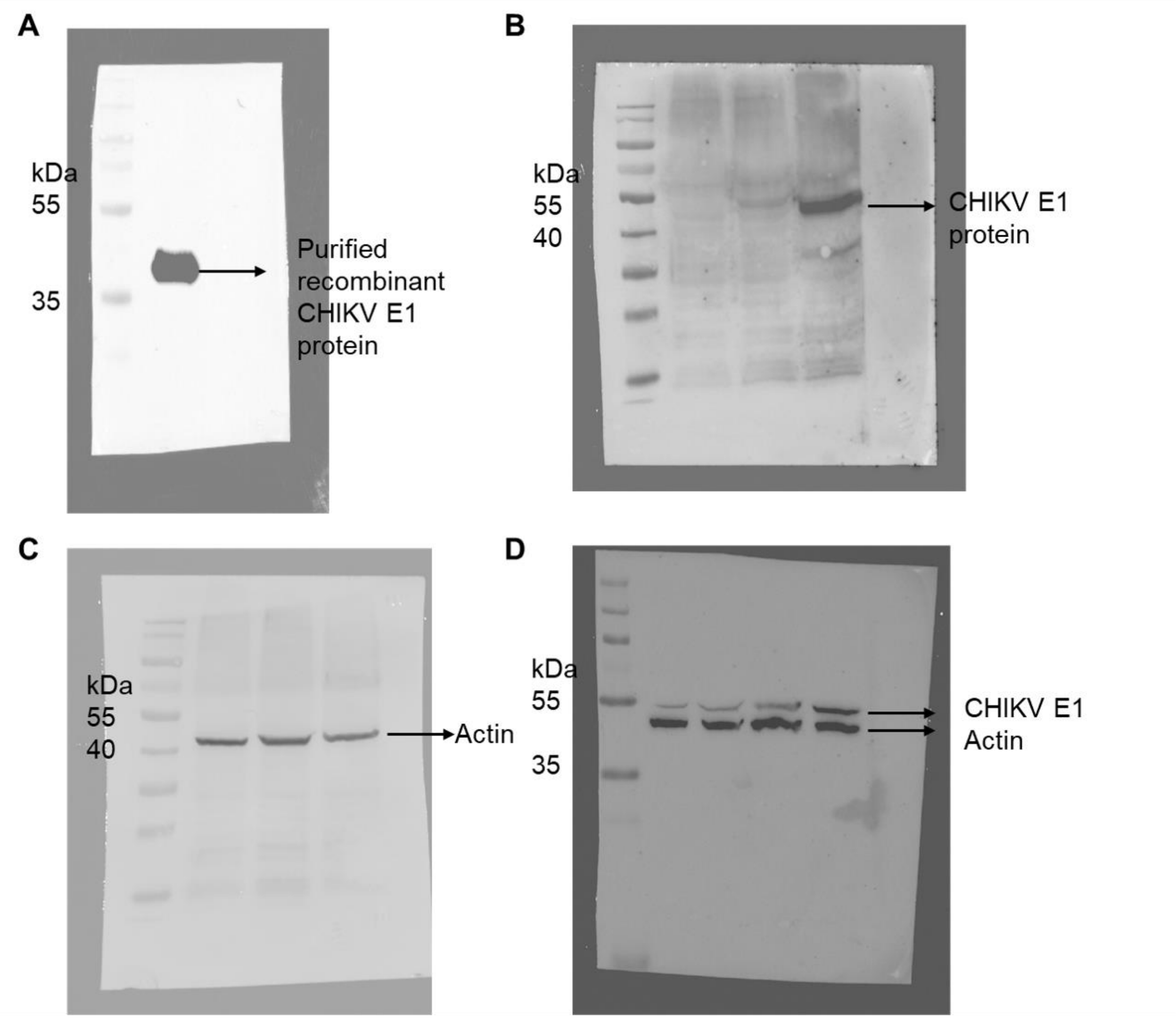

Figure S3. Validation of antibodies used in Western blots. A) Anti-His antibody (Santa Cruz, Cat. sc-8036-HRP) used to detect His-tagged recombinant CHIKV E1 protein cloned in pET29a. The dilution of 1:4000 was used in PBS+0.1% Tween-20 with 2.5% BSA; B) Anti-CHIKV E1 mice sera (in house raised in mice) used to detect envelop 1 protein from CHIKV infected lysate. The

dilution of 1:3000 was used in PBS with 2.5% BSA; C) Anti-actin antibody (sc-47778-HRP, Santa Cruz) used to detect actin protein. The dilution of 1:6000 was used in PBS+0.1% Tween-20 with 2.5% BSA; and D) Anti-CHIKV E1 mice sera and anti actin antibody was used to detect CHIKV E1 protein and actin protein from CHIKV infected lysate with same conditions as mentioned above. The proteins were resolved in 12% SDS-PAGE gel and transferred to nitrocellulose membrane.

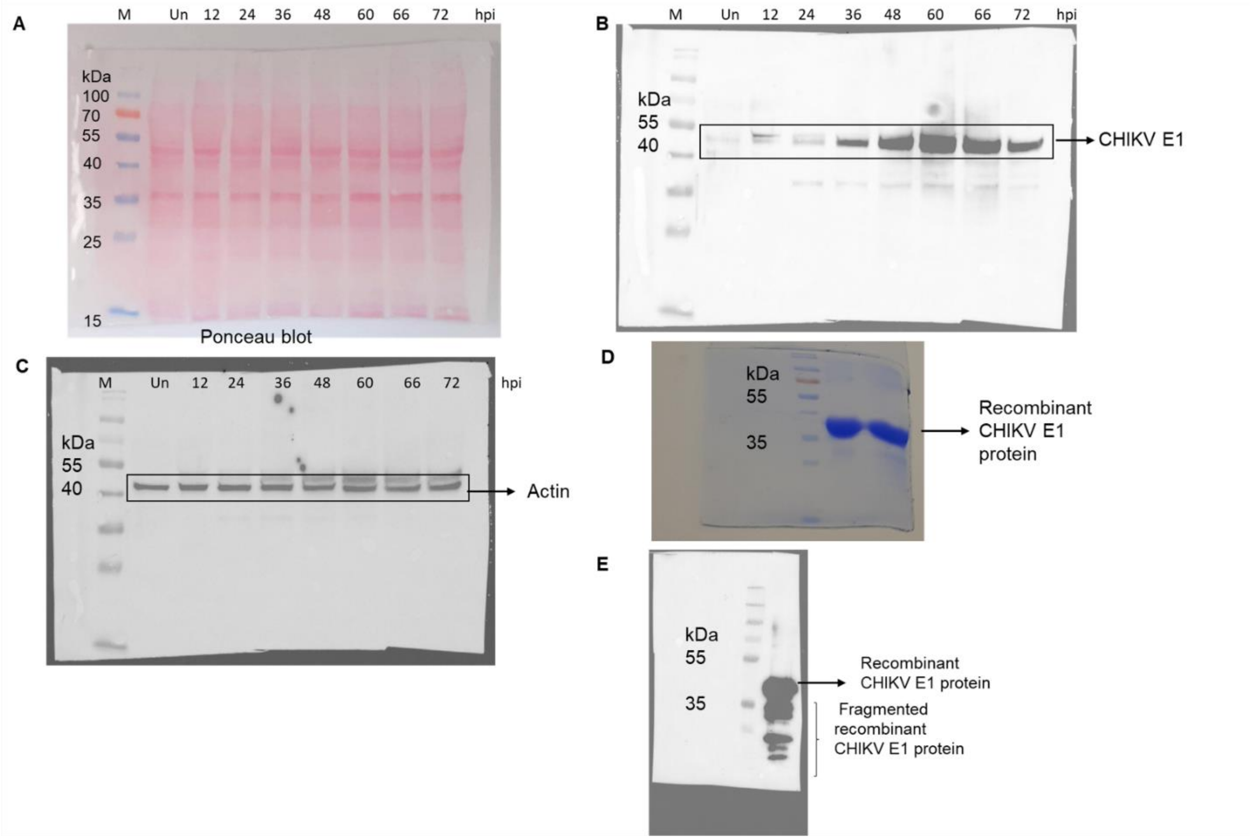

Figure S4. Raw figures of gels and western blots used in the manuscript. A) Ponceau stained nitrocellulose membrane showed in Figure 1C; B) Full image of CHIKV E1 blot showed in Figure 1C, and C) anti-actin antibody incubation of the same blot; and D) SDS-PAGE of purified recombinant CHIKV E1 protein and E) Western blot of purified recombinant CHIKV E1 with anti-His antibody.
